# The Evolutionary Psychology of Envy and Jealousy

**DOI:** 10.3389/fpsyg.2017.01619

**Published:** 2017-09-19

**Authors:** Vilayanur S. Ramachandran, Baland Jalal

**Affiliations:** ^1^Center for Brain and Cognition, University of California, San Diego, San Diego CA, United States; ^2^Behavioural and Clinical Neuroscience Institute, Department of Psychiatry, University of Cambridge Cambridge, United Kingdom

**Keywords:** evolutionary psychology, envy, jealousy, emotion, novel thereperutic technique, thought experiments

## Abstract

The old dogma has always been that the most complex aspects of human emotions are driven by culture; Germans and English are thought to be straight-laced whereas Italians and Indians are effusive. Yet in the last two decades there has been a growing realization that even though culture plays a major role in the final expression of human nature, there must be a basic scaffolding specified by genes. While this is recognized to be true for simple emotions like anger, fear, and joy, the relevance of evolutionary arguments for more complex nuances of emotion have been inadequately explored. In this paper, we consider envy or jealousy as an example; the feeling evoked when someone is better off than you. Our approach is broadly consistent with traditional evolutionary psychology (EP) approaches, but takes it further by exploring the complexity and functional logic of the emotion – and the precise social triggers that elicit them – by using deliberately farfetched, and contrived “thought experiments” that the subject is asked to participate in. When common sense (e.g., we should be jealous of Bill Gates – not of our slightly richer neighbor) appears to contradict observed behavior (i.e., we are more envious of our neighbor) the paradox can often be resolved by evolutionary considerations which h predict the latter. Many – but not all – EP approaches fail because evolution and common sense do not make contradictory predictions. Finally, we briefly raise the possibility that gaining deeper insight into the evolutionary origins of certain undesirable emotions or behaviors can help shake them off, and may therefore have therapeutic utility. Such an approach would complement current therapies (such as cognitive behavior therapies, psychoanalysis, psychopharmacologies, and hypnotherapy), rather than negate them.

Human emotions are very poorly understood even though there is a long venerable tradition of research pertaining to them, going all the way back to Darwin’s “Expression of Emotions in Animals and Men” (Darwin, 1872; on emotions see also Ekman and Friesen, 1971; Izard, 1977; Wierzbicka, 1986; Russell, 1994; Oatley and Jenkins, 1996; Fredrickson, 1998; Lewis, 2000; Panksepp and Biven, 2012). This is in stark contrast to research on such arcane topics as – say, apparent motion perception which has been studied in excruciating – sometimes pointless – detail. In truth our “common sense” understanding of emotions is probably closer to the mark than the insights offered by specialists working on the subject (just read a good Jane Austen novel).

One problem is that physiologists and psychologists who study emotions do not look at them enough from an evolutionary standpoint (for exceptions, see Nesse, 1990; Ekman and Davidson, 1994; Johnston, 1999; Cosmides and Tooby, 2000; Tooby and Cosmides, 2008). This is unfortunate because as Dobzhansky famously said, “Nothing in biology makes any sense except in the light of evolution.” This may seem obvious but even though people often pay lip-service to it, it is an attitude that has yet to permeate mainstream neurology and psychology [as championed eloquently by Tooby and Cosmides (1990)].

In evolutionary terms, emotions are adaptive responses to the environment that increase my chances of survival. But unlike simple adaptations – say the sensation of pain when my hand is pocked with a hot rod motivating me to withdraw it – emotions are much more complex. They orchestrate a more organized response. If I suspect a tiger is nearby, a fight/flight reaction (mediated by limbic structures) will activate several aspects of my physiology and cognition, each recursively feeding on the other – influencing my behavior (e.g., Nesse and Ellsworth, 2009).

It is of course possible to swing to the other extreme and assume that every little quirk of human behavior must have a module devoted to it that has direct survival value that was honed by natural selection; a view perpetuated by media accounts of evolutionary psychology (EP) (although the main proponents of this approach are usually careful to avoid such pitfalls, including David Buss, Donald Symons, John Tooby, Leda Cosmides, Melvin Connor, Christine Harris, and Steven Pinker). There are four pitfalls to watch out for in taking this approach: (1) Not everything is adaptive; many quirks of mind may be incidental byproducts or atavistic remnants of things that were once useful (the psychological equivalent of the vermiform appendix). (2) The second pitfall is that many so-called universal psychological traits may be learned; people may have “converged” on the same solution to similar environmental challenges; e.g., cooking is almost universal but we do not postulate a cooking-module in the brain hardwired through natural selection. It was probably based on the accidental discovery of game roasted by forest fires. (3) Both the observed trait and its explanation are banal; obvious even to your grandmother. For example, men like young women with clear skin and big breasts because they are more fertile; so the genes that predispose to such preference would multiply. Is there anyone who doesn’t know this? (4) When the explanation is not banal then it is often very difficult – almost impossible – to test experimentally or refute. It fails to fulfill the falsifiability criterion of Popper. A good example is the satirical theory one of us proposed to account for why “gentlemen prefer blondes” (Ramachandran, 1998), suggesting that they do so because it allows them to detect early signs of parasitic infestation and aging – both which reduce fertility. In addition, reciprocity of sexual interest is more obvious in blondes because of the dilatation of dark pupils against a pale iris; as is the pink flush of orgasm in a light skin – which lowers the chances of cuckoldry and increases likelihood of implantation. (Blushing, too, is more obvious in blondes who in effect is sending an involuntary “truth in advertising” signal conveying that she can’t cucold with impunity without the blush of embarrassment giving her away.) This account was taken seriously by some members of the EP community; indeed, we concede the possibility that what began as a satire may have more than a grain of truth in it! An even more far-fetched theory was proposed jocularly by my colleague JA Deutsch (personal communication). Deutsch suggests that the reason women experience nausea and vomiting early in pregnancy is because “the odor of vomitus” would discourage the husband from having sexual intercourse with her. This makes evolutionary sense given the known risk of abortion resulting from intercourse in such cases. As a final example, let us suggest that the reason we flock to aquaria is that our Devonian piscine ancestors were attracted to other fish and we have remnants of this affinity fossilized in our brains. It is easy to see the absurdity of these three examples, but some EP arguments have the same form.

Where EP is on firm ground is when it avoids as many of these pitfalls as possible. Then it becomes fun to explore. The name of the game is to make observations of human psychology that initially seem surprising, counterintuitive, and apparently non-adaptive and then go on to show there might be a hidden evolutionary agenda. This strategy does not necessarily prove the theory but it makes it more credible than if the pitfalls had not been avoided. We would like to illustrate this approach by considering the very simple example of jealousy or envy. These two words are used interchangeably in the United States, but in the United Kingdom the former is more often used in a sexual context, the latter in other contexts. In either case the “target” is usually someone who is perceived to be better off in some respect than you or whose access to resources is better than yours. Jealousy also has a possessive component; I want to actually deprive the other person’s resource and claim it as my own. It is a negative emotion. Envy is not quite as negative – it does not have the same sharp edge and it motivates emulation to gain independent access to similar or even better resources. The extreme along the same spectrum would be pure admiration of someone, who, through inborn talent and intense effort, is better off than you. I am envious of my neighbor who got an award from the local mayor, but I admire Francis Crick.

Jealousy is a motive of immense potency. Although you are often consciously aware of being jealous or envious of someone, sometimes the actual reasons for the envy are buried in your unconscious and disguised by rationalizations. Ironically, what you really value in life is more often revealed by asking yourself who you are jealous of rather than asking yourself directly “what do I value.” The latter often taps into what society expects you to value; your “superego” takes over – and you are aware only of what you should want rather than what you really want. Envy and jealousy, on the other hand, kick in as a gut reaction in your emotional/evaluative system long before you become conscious of it.

Introspection is unfashionable in contemporary psychology largely due to the lingering effects of behaviorism. Contrary to this view, we will argue (and demonstrate in this paper) that introspection (if accompanied by cross-validation across other thoughtful subjects) can be a valuable source of insights into the internal logic and evolutionary rationale of certain complex emotions like envy. Obviously, objections can be rightfully raised against the – purely subjective – exercise of introspection, which is why it is imperative to eventually test these conjectures by making counterintuitive predictions that can be empirically falsified (using a rigorous scientific approach). But meanwhile one can have fun speculating on possibilities.

The central argument in this paper is that one can achieve a deeper understanding of emotions by introspective “thought experiments”; asking yourself – and others – which social situation (A or B) would make you more prone to that emotion and what the environmental triggers are. One can then construct meaningful evolutionary scenarios as to why a particular trigger (A) might have evolved to produce a given emotion even though common sense might dictate that another trigger (B) should be more effective. For the more flagrant emotions (like aggression) the triggers and their evolutionary rationale are obvious and probably mediated by limbic structures in the brain. But more complex emotions require more complex triggers (or combinations of them) to elicit them. The evolutionary logic of these emotional triggers may not be obvious at first but can be teased apart – by imagining yourself in certain situations and simply asking yourself how you would feel. Most complex emotions may depend strongly on social interactions, context, self-worth evaluation, and a sense of who you are as perceived by others. Examples would include pride, arrogance, superciliousness, ambition, guilt, gratitude, and jealousy (the topic of this article). Unlike basic emotions like aggression and fear – mediated mainly by the limbic fight/flight response, these more complex emotions probably require interactions with the orbitofrontal cortex. Such emotions, including the ability to introspect on them (“I am jealous because, etc.”) are probably unique to humans or especially well developed in us. They may require the construction of a “meta-representation” – a representation of earlier representations in the brain (knowing that you know, or knowing that you are jealous).

It raises an important issue. Do the subtler emotions (like pride, ambition, envy, and guilt) each have a peculiar flavor. For example, does jealousy have its own unique subjective qualia or is it a vague nebulously negative feeling that becomes either tinged with its unique “flavor of jealousy,” or is it merely inferred *post hoc*, based on social context? Unlike (say) the quale of red which kicks in right away independent of social context? Introspection suggest the former; for we often catch ourselves experiencing a twinge of jealousy of a friend – often with surprise and embarrassment – before inferring the reasons, context, etc.

We would venture that a frontal patient may still be capable of aggression, fear, and lust but not of envy or romantic love (which have complex and subtle social dimensions). Such a patient will have great difficulty introspecting on his own emotions – not just expressing them. In evolutionary terms, it is worth noting that even though emotions are privately experienced almost all of them are meaningless except in relation to others; i.e., in a social context (e.g., envy, pride, jealousy, and kindness). This is partially true even of the more basic emotions such as fear, lust, anger, and pain; for instance, we shout “ouch” to attract attention.

What triggers jealousy, beyond the obvious of someone who is better off? And can the functional logic of these triggers (or peculiar combination of social cues) be explained in evolutionary terms; i.e., what might be their survival value? Through introspecting on ourselves and through informally surveying friends, students, colleagues, etc., we composed the following list. For each item on the list, we will try to come up with a plausible evolutionary scenario. Especially important is the question of why you make a particular choice even though common sense might favor the other choice. We would emphasize that these are at this stage merely preliminary informal surveys, whose goal is to prompt further inquiry using rigorous methodology to collect formal data. (In the study of visual perception, analogously visual illusions have a long and venerable tradition in making important points long before detailed measurements were made to confirm those points).

(1) Are you more envious of: (A) someone who is similar to you in most respects but is a bit wealthier (say 50% wealthier) or (B) more envious of Bill Gates? Is a beggar jealous of a slightly more successful beggar or of Bill Gates?

The answer is almost always the former (10 out of 11 people we surveyed chose A). This does not make sense. One usually expects the strength of an emotion to be directly proportional to the resource being sought after; e.g., blood glucose determines the degree of hunger. Following this argument, shouldn’t you be more envious of Bill Gates? Common sense might dictate that the better off someone is than you are, the more envious you should be. But counterintuitively this isn’t true. “Common sense” (the logical or reasoning part of the brain) of course also arose through evolution – but arguably for different needs; i.e., abstract generalizations such as rules of logical inference – which have only limited access to the “laws of emotions” (keeping in mind the modular architecture of the human brain). You ought – logically – to be more jealous of Bill Gates because he has more resources. But the “emotion module” is wired-up for immediate “gut-reactions” like jealousy, sometimes overriding logical inferences. In general, gut-reactions and the “rationality faculty” deliver consistent answers – but not always.

Where conventional EP theories sometimes fall short is that they aren’t always counterintuitive. For example, they “explain” that men prefer younger women because they are more fertile. Neither the phenomenon itself (the choice of younger women) nor the standard explanation (“they are fertile”) is counterintuitive. They fail to fulfill what we call the “grandmother test” – what your grandmother might have deduced from the mere application of common sense. The trouble is that in many scenarios commonly considered in EP, these two (common sense vs. hidden evolutionary agenda) make the same prediction; the only way to dissociate them is to create highly contrived scenarios; which we shall attempt, in this paper.

What is the evolutionary logic that drives envy; e.g., the fact that you envy your neighbor more than Bill Gates? The answer is that the whole purpose of envy is to motivate you into action either by independently trying harder (envy) or by coveting and stealing what the other has (jealousy). This is why jealousy has an aggressive component, but envy is more positive sometimes even being tinged with admiration.

Turning to Bill Gates vs. a more prosperous beggar, we believe this can be explained quite readily by the axiom that envy evolved to motivate access to resources that are in demand by others in your group. If I am the poor beggar my brain quickly computes that in all likelihood the very rich Gates is either deservedly much richer (i.e., he is far smarter), or just extremely lucky. Evolutionarily speaking, there is no point in being jealous of him because he is “off scale” either in ability or luck, so no amount of effort by me can result in reaching his level of prosperity; envy would motivate an inappropriate and futile waste of resources. The richer beggar, on the other hand, may be only slightly smarter, luckier, or more hardworking than me, so there is some chance, at least, that envy might motivate me to exceed his access to resources (or jealousy might make me steal it away from him with impunity).

(2) Are you more envious of (A) someone equal to you in talent, effort, etc., but he/she gets undeservedly promoted over you or (B) of someone who is genuinely better than you, who is rewarded?

The answer is almost always “A” (11 out of 11 people we surveyed chose A). Again, note that this makes no obvious sense; if you want to be as rich as your neighbor, what does it matter whether he was undeservedly rewarded or legitimately rewarded. This can be teased apart further. For example, does it matter whether the other guy got rewarded by the boss, (A) because he is (naturally) genetically more intellectually gifted than you, (B) more hard working, or (C) arbitrarily for no reason? EP would predict that you would be most envious of “B” because the envy would motivate you to do something about it, whereas competing with “A” might be futile; you cannot over-ride genetic endowments. (You might be angry at the boss for being unfair, but not jealous of the recipient.) “C” would make you envious too if “doing something about it” includes complaining to your boss. The greater envy for “B” over “A” should be especially true if “A” is vastly better endowed than you genetically; if he is only slightly better endowed then some envy would be useful – motivated hard work can help you to overcome genetic limitations. This would be analogous to the beggar vs. the other beggar scenario provided above. The third scenario (C) would not provoke envy; it would provoke anger toward the person who unfairly rewards your neighbor. In short, we can show that even though a surface-level analysis of a human psychological propensity makes it seem maladaptive, there is often an evolutionary hidden agenda that drives that propensity, and makes it comprehensible. We are not making a definitive argument here but hopefully providing food for thought.

(3) Let us say I were to prove by brain scans or some other reliable measure (e.g., mood/affect inventory) that (A) the Dalai Lama was vastly happier on some abstract, but very real, scale than (B) someone (say Hugh Heffner) who has limitless access to attractive women. Who are you more envious of?

Most men are more envious of the latter (9 out of 9 males we surveyed chose B). In other words, you are more jealous of what the other person has access to (in relation to what you desire), than of the final overall state of joy and happiness. This is true even though common sense might dictate the opposite. Put differently, evolution has programmed into you an emotion (jealousy) that is triggered by certain very specific “releasers” or social cues; it is largely insensitive to what the other person’s final state of happiness is. The final state of happiness is too abstract to have evolved as a trigger of envy or jealousy.

For similar reasons, if you are starving it makes more sense that you would be more jealous (at least temporarily) of someone enjoying a fine meal than someone having sex with a beautiful woman or man. If you are only slightly hungry, however, you might pick sex. This is because there is an unconscious metric in your brain that computes the probability of finding food in the near future vs. finding a nubile, available mate; and of the urgency of your need for food over the urgency of mating. If you are starving to death and have one last fling, you have only that single mating opportunity whereas if you eat and live you will have plenty of mating opportunities in the future.

(4) Imagine a scenario (A) in which you see another guy/girl making love to a woman/man you are attracted to and desire. You are jealous. But what if (scenario B) you see the same guy/girl having even more passionate sex with a woman/man you are not attracted to.

Surprisingly, you are more jealous of him/her in “A” (13 out of 15), even though one might expect the answer to be “B” – i.e., you should be jealous of and strive to achieve – his final pleasurable state (B) than what leads up to it (A). Again evolution prevails over common sense in a very specific manner. You have a metric in your head (your assessment of your own attractiveness constructed unconsciously by monitoring the frequency and “objective beauty” of other women who were attracted to you in the past) of what you want and are capable of. These triggers determine who or what you are jealous of, even though it doesn’t make any sense. The situation is not fundamentally different from you eating cotton candy. Even though you know rationally it is not good for you, these “carbohydrate binges” were wired into your brain during prehistoric times when food was scarce to help tide over dry spells of famine. In the case of food preference, this idea might seem obvious (although it wasn’t obvious to us until Steven Pinker spelled it out). But in the case of more complex emotions like jealousy, the idea has not been adequately explored in the manner attempted in this article. The general idea is that even complex and subtle nuances of a certain emotion can be analyzed in this manner.

(5) Another example also illustrates how some emotions despite being counterintuitive and seemingly illogical initially reveal a hidden evolutionary agenda. (A) You see your neighbor (who is similar to you in most respects) having moderately enjoyable sex with a woman whom you moderately covet; (B) you see two ugly tramps having intensely pleasurable sex with each other. Who would elicit more envy?

Again, for reasons already alluded to, most people are more envious of “A” (12 out of 15 people we surveyed chose A). This is another example of being envious, not of the final level of intense pleasure (as one might naively expect) but of someone having access to – and only slightly enjoying – something for which you have a modest desire and will only modestly enjoy (but access is denied). Thus, we see that what triggers envy are certain social cues; “happiness” is too abstract to be envious of. All this seems plausible but – once again – we emphasize the need for caution in interpreting such data. You might avoid choosing the tramps not because of the evolutionary reasons alluded to above but because any association with tramps elicits avoidance.

In general, the less complicated or contrived the thought experiment, the more straightforward the result and the interpretation. The simplest example of the genre of thought experiments discussed so far would be; would you be more jealous of (A) your neighbor who is slightly smarter than you who gets a huge raise and award for her performance – or (B) you and she each buy a lottery ticket and she ends up winning 500,000 dollars? If you introspect, your answer like that of most people might be that you would be less jealous in the second scenario (lottery) because you recognize that no amount of extra motivation from you (driven by jealousy) could repeat a fluke accident.

We now introduce the concept of “relevant social circle”:

(6) Imagine you are a first generation Indian immigrant in the United States; (A) your neighbor is also an Indian immigrant of comparable talent; (B) a Chinese immigrant; (C) an American local. Say “A” has something you covet and you envy him; “B” has the same thing and “C” does too. Who would you be most envious (jealous) of? Let us say for the sake of argument that what all of you covet is a woman or man.

Most would envy “A” more than “B” or “C” (11 out of 11 we surveyed chose A). It is the unconscious metric again. Your brain says (in effect), “A” has had the same privileges, opportunities, status, etc. as me, so there is some point in my being envious of him in order to motivate me, since I have at least some chance of gaining access to the same resources; he provides an “existence proof” that someone who is very similar to me can have access to the same resource. “B”, on the other hand, is a complete unknown. Finally, “C” may be favored for completely arbitrary reasons such as racism and xenophobia against a member of the more privileged majority White American culture. Unlike “A”, “C” has most likely had more privileges and opportunities throughout his life, so there is not much motivation for you to compete, as you don’t have a chance of gaining access to his resources – thus envy would lead to a futile waste of resources and time. Instead, the evolutionary consequence might be anger toward “C”, rather than an attempt to compete and balance the inequity through personal effort (you wouldn’t be jealous). Obviously, in many situations the two emotions overlap.

(7) Geographic proximity; this is, a special case of the “relevant social circle” effect: Compare the two cases: (A) Joe lives in Timbuktu. He is very similar to you in talent, looks, capacity for work, etc. but he is twice as wealthy as you; (B) Joe is your neighbor and twice as wealthy.

Most people would be envious of “B” more so than “A” (11 out of 11 we surveyed chose B). Again this makes evolutionary sense. There are millions of people who are like “A” and even though I have assured you logically that they are identical to “B”, your brain requires more direct triggers. On the other hand, you see and interact with “B”, and this is a direct trigger for envy to kick in. “A” is simply too abstract to relate to and, more importantly, is not competing for the same resources as you (and is, in any event, too far from you to do anything about). There would be no motivation to work harder since even if you did, you would not gain access to resources in Timbuktu. You might admire him – even emulate him – from a distance, but it would be futile to be envious.

(8) There is a very attractive woman (or man) you have your eye on and have reason to believe that you are within her range of acceptability and is attracted to you. But another man walks in and she is instantly attracted to him and walks off with him. He is one of three people: (A) he is stunningly handsome and wealthy and walks off with her; (B) only slightly better looking and handsome or even identical to you in most respects; and (C) an ugly old poor tramp. Who would you be most jealous of?

Most people are jealous of “B” (8 out of 11 people we surveyed chose B). After “B” we suggest that people would be jealous of “A” and then “C.” The reason we suggest this is that as a motivator of action, jealousy would be most likely to be effective in situation “B.” Jealousy might be futile in “A,” and you can’t blame him (or her) in succeeding in tempting her away. Lastly, the behavior of your potential target mate in “C” – her choice of an ugly tramp – suggests that her choices are completely idiosyncratic and unpredictable. So there is not much point in even trying.

Jealousy for siblings is a special case. Since a sibling [whether identified correctly or misjudged to be a sibling as a result of close proximity from early childhood (i.e., “the kibbutz effect”)] shares half your genes you should theoretically be less jealous of his access to resources than you would be of a complete stranger. But this is complicated by the fact that you are in direct competition for the same food resources (e.g., during weaning) delivered by parents and by the “relevant social circle” phenomenon predicted above. The net result would be a complex hybrid of emotions, as is often indeed the case with siblings. For the same reason, we would predict that jealousy of parents should be a rarity.

We reiterate that the data presented here are merely preliminary surveys rather than derived from formal research. While introspection can be a valuable source of insight as a starting point, these conjectures must eventually be tested using empirically sound methods. What we have introduced is just the bare skeleton outline of the evolutionary logic that might be driving jealousy. There are bound to be other complex contextual and personality variables that influence any particular individual’s choice in each of these far-fetched scenarios. Nevertheless, these speculations might provide a starting point for a more sophisticated understanding of jealousy than has been hitherto possible, taking us well beyond our “common sense” understanding of this complex human emotion. We believe a similar strategy could be applied for understanding other equally complex human emotions. The goal is to seek the “ulterior motive” in strictly evolutionary terms, of forms of behavior that might initially seem inexplicable. Conversely, pointing out that men like big breasts of young women simply doesn’t cut it; EP does not tell you anything that common sense doesn’t [see Konner (2015) for an elegant exception].

We have considered only one example, namely, jealousy – to illustrate our strategy but, obviously, one could apply it to other emotions. Consider embarrassment, for example. If you are a man buying an adult erotic DVD, would you be more embarrassed if the sales clerk at the cash register was: (A) a handsome young man; (B) a beautiful woman; (C) an old lady; and (D) an ugly old man? The answer is usually “B” or “C” (11 out of 13 males chose either “B” or “C”) but why? And might not the answer give us novel insights into the evolutionary origins of embarrassment?

Another intriguing question is what kinds of triggers (or combinations) elicit jealousy (which has a sharp edge) as opposed to admiration, other than the fact that the latter elicits emulation without malice, whereas the former motivates depriving the target or resources you wish to acquire. If there are two prizes being offered in a competitive game of skill – one of which the target person has acquired – the result might be admiration motivating parallel acquisition of the remaining prize. On the other hand, if there is only one resource, aggressive acquisition requires motivation via jealousy.

A recurring theme, throughout this essay is the contrast between the “rational” or intuitively “obvious” view, on the one hand, and the actual emotion experienced, on the other; e.g., we suggest that based on common sense, beggar “A” should be more jealous of Bill Gates than of beggar “B.” One might initially expect that since jealousy is motivated by the discrepancy of resource, the larger the discrepancy the more the jealousy, but as we have seen that is not the case. The reason for this again is that it would be a poor strategy, evolutionarily, for a beggar to seriously allocate resources of time and energy to become equal to Bill Gates, when the same resources could be more profitably allocated to the more realistic goal of competing successfully against a neighboring beggar.

As another analogy of the difficulty disentangling different threads of culture, genes, emotion, logic, etc., consider the case of you being jealous of your girlfriend having a fling with a man vs. a woman (assuming she is not a habitual lesbian). Most men in our experience would be more upset by the former. This could be for the obvious reason that a man does not want her to be accidentally inseminated and cuckolded, whereas there is no danger of this with a lesbian fling (again, common sense ought to predict that you ought only to be jealous of the fling that produced more pleasure; the gender of the fling-partner should be irrelevant). An alternative to the EP counter-cuckolding argument would be that the male is regarded as being in an “equivalent class” and elicits a bigger competitive jolt – hence jealousy – than a fling with a woman.

Given how primitive our knowledge of the subtle nuances of human emotions is, it is hardly surprising that our insight into the causes of mental illness – which are primarily disturbances of emotions – is equally primitive. It can hardly be true that all the diverse emotional disturbances of a complex organ like the human brain fall into a handful of categories; mood disorders, psychotic disorders, dissociative disorders, etc. (leaving aside the many hundreds of bogus disorders fabricated to be included in the DSM for insurance purposes).

We believe that a deeper understanding of the functional logic and emotional disturbances that underlie mental illness could be obtained by adopting an evolutionary perspective. As we have discussed in more details elsewhere (see Jalal and Ramachandran, in preparation), we propose that gaining deeper insight into the evolutionary rationale of negative emotions and behaviors (including primitive psychological defense mechanisms) could have therapeutic utility. This approach places emphasis on the evolutionary origins of mental quirks – including pathologically amplified ones – which makes it different from conventional therapeutic techniques. It merges elements of psychoanalysis (by stressing the role of primordial drives and defense mechanisms) and cognitive behavior therapy (CBT; in that it takes the patient step by step through logical “what if” questions) – embedded in an overarching evolutionary theme. Among other strategies, it uses the approach of posing absurdly farfetched dilemmas to get to the axiomatic system of values (and their derangements) that drive your behavior. A striking example is the observation that noticing what or who you are jealous of is often a more “honest” and accurate indicator of what you truly value than simply asking the same question directly to yourself. Once such a deeper understanding is gained you can begin to shake off emotions and behaviors that are overall maladaptive in the current context. This therapeutic approach would supplement rather than negate current psychotherapies and pharmacological approaches.

## Ethics Statement

The study included informal surveying of friends and colleagues, and as such was exempt from ethical approval.

## Author Contributions

All authors listed have made a substantial, direct and intellectual contribution to the work, and approved it for publication.

## Conflict of Interest Statement

The authors declare that the research was conducted in the absence of any commercial or financial relationships that could be construed as a potential conflict of interest.
